# Accelerated neurostimulation protocols for auditory hallucinations: a systematic review and meta-analysis

**DOI:** 10.3389/fpsyt.2025.1491487

**Published:** 2025-06-13

**Authors:** Tremearne Hotz, Natalia Kosyakova, Manu Sharma

**Affiliations:** Department of Psychiatry, Institute of Living, Hartford, CT, United States

**Keywords:** transcranial magnetic stimulation (TMS), theta burst stimulation (TBS), accelerated, auditory hallucinations (AH), psychosis

## Abstract

**Objective:**

To explore the efficacy and characteristics of accelerated (more than once daily) protocols of repetitive transcranial magnetic stimulation (rTMS) and transcranial Electrical Stimulation (tES) in treating auditory hallucinations (AH) and other psychotic symptoms.

**Methods:**

”We searched Pubmed” using relevant MeSH terms and keywords to identify relevant literature. Standard mean difference (SMD) and 95% confidence interval (CI) values were used to evaluate the effects of rTMS and tES.

**Results:**

Eighteen studies were included, eight which used rTMS and ten which used tES. AHs and positive psychotic symptoms (PPS) improved in all studies from before to after treatment (SMD = 0.64, 95%CI = 0.77 to 0.51). Superiority was seen in the groups using fMRI guidance and using cTBS. Thirteen studies used a sham group as a control, which collectively showed statistically significant improvement in AHs with a moderate effect size (SMD = 0.34, 95%CI - 0.50 to 0.18). However, these studies included a high level of heterogeneity as measured by Cochran’s Q and I^2^. Meta-analysis performed showed no consistent improvement of negative symptoms and did not differ significantly between the treatment and sham groups.

**Conclusion:**

There appears to be a therapeutic effect for accelerated neurostimulation protocols for AHs on par with non-accelerated approaches. These protocols take up less overall time and often provide less overall stimulus. This result needs to be confirmed by large-scale randomized controlled trials before this finding can be recommended in clinical practice.

**Systematic review registration:**

https://osf.io/69azy/, identifier 10.17605/OSF.IO/69AZY.

## Introduction

The treatment of Schizophrenia and Schizoaffective Disorder poses a significant challenge to psychiatry. Despite advancements in psychiatric medication, which remains the primary treatment approach, efficacy often extends only to alleviating positive symptoms and leaves nearly a third of patients with partial or no relief (1, 2). Furthermore, pharmacological interventions often entail therapy that lasts years or even is lifelong, increasing the burden on patients for side effects like extrapyramidal symptoms and metabolic syndrome. Acknowledging this therapeutic gap, non-pharmacological interventions such as Transcranial Magnetic Stimulation (TMS) and Transcranial Electrical Stimulation (tES) are gaining prominence in both research and clinical practice, offering promising alternatives for patients with these conditions (3).

rTMS and tES are safe and non-invasive techniques that utilize alternating magnetic fields and electrical currents respectively, to modulate electrical currents in the brain (4). TMS, uses electromagnetic pulses to create an electrical field in specific areas of the cortical brain parenchyma. This can either stimulate or inhibit the neuronal activity, depending on if the stimulus is high frequency (ie 20Hz) or low frequency (ie 1Hz) respectively. When used repetitively, as in rTMS, many pulses of electromagnetism are provided in ~30 minute sessions, typically daily, over the course of many weeks. More recent research has utilized theta burst stimulation (TBS), in which higher frequency (50 Hz) bursts are provided intermittently to mimic endogenous theta wave stimulation.

tES instead utilizes a constant weak electric stimulus either with direct (Transcranial Direct Current Stimulation or tDCS) or alternating (Transcranial Alternating Current Stimulation or tACS) currents to achieve a similar goal of effecting cortical excitability (5). Both TMS and tES approaches have shown promise in the treatment of conditions like depression as well as the positive and negative symptoms of psychosis (6–10). For auditory hallucinations (AH), targeting of the Left Temporoparietal Junction (L-TPJ) consistently has shown improvement in patients already being treated with antipsychotic medication (11, 12).

Recent research has introduced accelerated protocols, where tES or rTMS sessions are administered multiple times per day. This approach has the advantage of delivering more bursts over a shorter time period. In conditions like Major Depressive Disorder, condensing TMS protocols which are typically several weeks down to as little as five days, achieved faster and stronger treatment outcomes (9). Accelerated protocols offer the practical advantage of requiring less time commitment compared to standard protocols, potentially improving the practicality and adherence patients have with these interventions.

The benefit of accelerated protocols for psychotic symptoms is still unclear. While previous meta-analyses have suggested a dose-dependent relationship for both rTMS and tDCS, the benefit of more stimulation appears to diminish beyond a certain point (10). This is similar to the dose response pattern observed for antipsychotic medication (13), suggesting that more treatment cannot be assumed to yield more response. Therefore, there is a clear need to determine the efficacy and feasibility of accelerated approaches for psychotic symptoms.

The present study examines the array of accelerated neurostimulation protocols employed in addressing psychotic symptoms, in particular AHs. Focus has been made to draw attention to differences in scheduling, stimulus location, frequency of stimulus, and total volume of stimulus given.

## Methods

### Selection of studies

This protocol was registered at the Open Science Foundation (doi: 10.17605/OSF.IO/QGUR7). A literature search was conducted in the PubMed for studies published or accepted for publication in the period between January 2014 and June 2024. This time frame was chosen to better reflect contemporary stimulation protocols and reduce heterogeneity. One database was explored due to limitation of resources. Cohort Trials and RCTs were included. Case studies and case series were excluded. Study screening was done by authors TH and NK and then compared. Any discrepancies were resolved through discussion. The following phrase was used in the search: (((Transcranial Magnetic Stimulation) OR (Theta burst stimulation) OR (Transcranial Alternating Current Stimulation) OR (Transcranial Direct Current Stimulation))) AND ((psychosis) OR (psychotic disorders) or (hallucinations)) AND ((accelerated) OR (twice daily) OR (Three times daily) OR (two times daily) OR (Multiple Sessions per day) OR (two sessions per day) OR (Three sessions per day) OR (Multiple times daily) or (intensive) or (rapid) or (high frequency)). The language was restricted to English.

### Eligibility criteria

**Figure 1:**
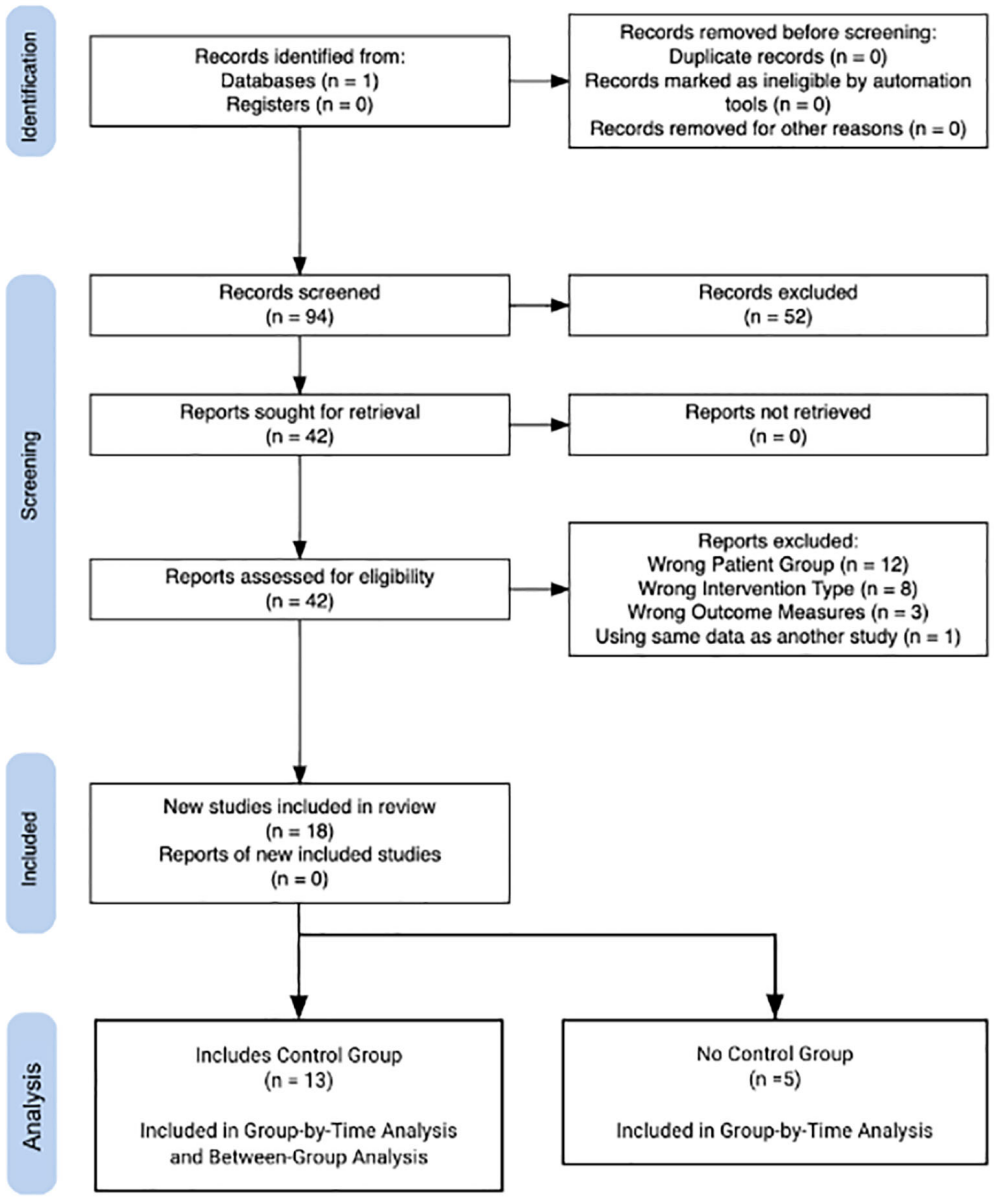
PRISMA flow diagram of study selection.

Two reviewers independently completed the literature search and evaluated the studies for inclusion in the study. The following inclusion criteria were used to select articles for inclusion in the present meta-analysis:

1. The study was a Cohort trial or Randomized Control Trial.
2. Patients were diagnosed with schizophrenia or schizoaffective disorder. Diagnostic criteria were based on DSM IV or V criteria.
3. Subject ages were over 18 years old.
4. Study reported positive psychotic symptom severity using standardized scales both before and after intervention.
5. Study used the intervention of TMS or tES more than once daily (**Figure 1**).

### Data extraction

The following information was extracted from eligible studies by one of the present authors (Hotz): publication year, frequency and location of treatment, characterizations of stimulus provided, number of sessions, total stimulation, type of coil, percentage of the individual motor threshold, and outcome measure. Auditory Hallucination Rating Scale (AHRS) was used as the primary outcome measure; if it was not obtained, the Auditory Hallucinations Subset of the Psychotic Rating Scale (AH-PSYRATS) score was used if available. The third choice was the scores for positive items of the Positive And Negative Syndrome Scale (PANSS). The scores for negative items of the PANSS (PANSS-N) were also used as an outcome measure.

### Statistical analysis

Weighted standard mean difference (SMD) and 95% confidence interval (CI) values were estimated to assess the effects of TMS and tES on AH using the Mantel–Haenszel method with a random-effects model. This model assumes different underlying effects, considering both within- and between-study variations, offering the advantage that it accommodates diversity between studies and provides a more conservative estimate of the assessed effect. The SMD was calculated using Cohen’s d. The weighted effect sizes were calculated for group-by-time and between groups for those studies that were controlled. The group-by-time analysis used change in symptom severity between post- and pretreatment for each study. The between group analysis used mean change between the treatment and sham groups. This method has been used in other similar studies (6).

**Figure 2:**
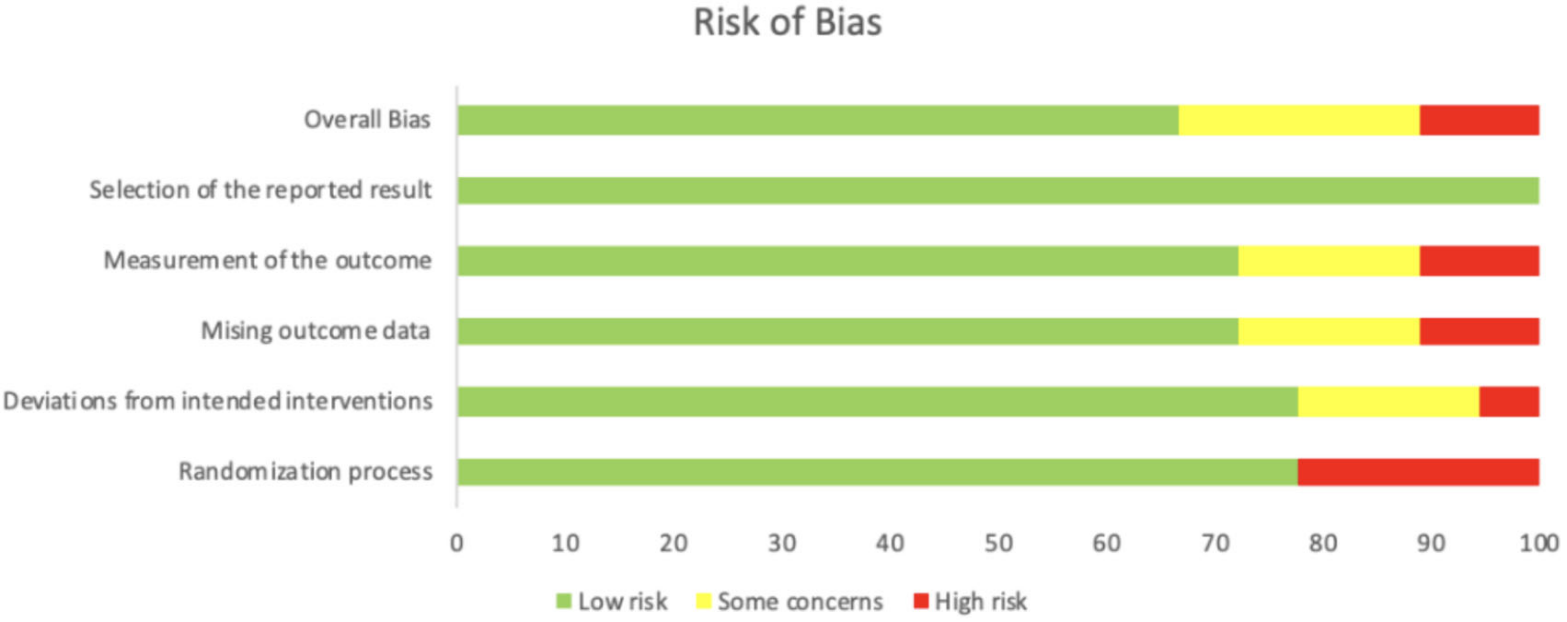
Cochrane risk of bias assessment summary.

**Figure 3:**
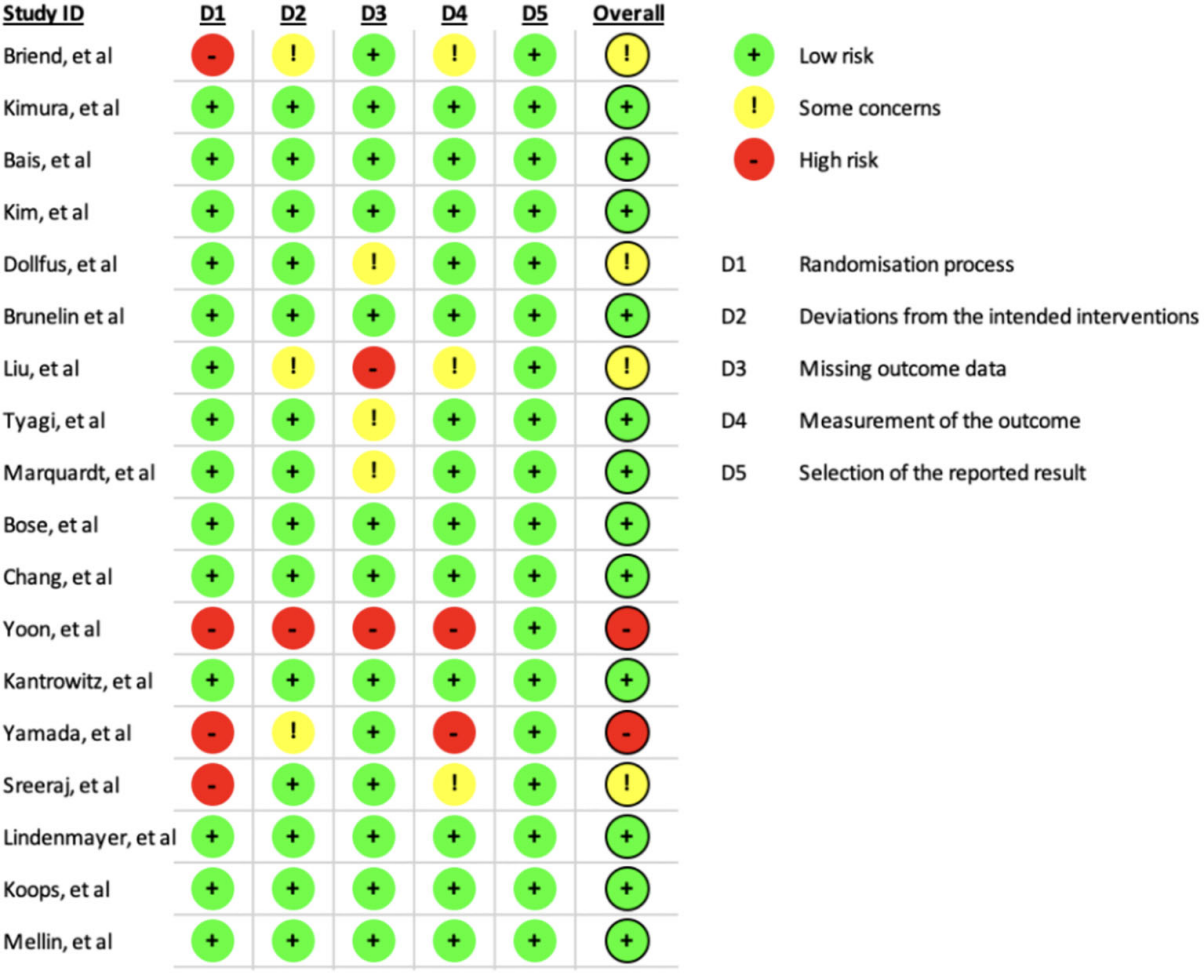
Cochrane risk of bias assessment.

The presence of heterogeneity was assessed using Cochran’s Q statistic and quantified using the I (2) statistic. A P value of Cochran’s Q-statistic of <0.1 or an I 2 value above 50% indicates the presence of a very high degree of heterogeneity. Risk of bias was evaluated using the Cochrane Risk of Bias Assessment (**Figures 2**, **3**).

## Results

### Study characteristics

The electronic search of Pubmed yielded 94 potentially relevant studies which were narrowed to 18 studies after evaluation with the inclusion criteria. Within these studies there were 24 distinct and eligible treatment groups. The basic information about these treatment groups is listed in **Table 1**. Overall, 11 treatment groups used TMS protocols and 13 used tES protocols. Of the excluded studies, 12 studies were excluded for not being the chosen patient population. 8 were excluded for being the wrong intervention. 3 were excluded for not having Auditory hallucinations as an outcome measure. One study was excluded for having the same data as another study which was included (14, 15).

**Table 1:** Characteristics of included studies.

Author	Year	n	Study Type	Intervention	Localization	Hertz	MT %	Days of treatment	Sessions per day	Total Pulses Delivered	Intensity (mA)	Total intervention Time (Seconds)	Outcome Measurements
H. Kimura, et al. (16)	2016	16	RCT	rTMS	LTPJ	20	80	2	2	10400		52	AHRS
F. Briend, et al. (18)	2017	11	Cohort	rTMS	fMRI LSTS	20	80	2	2	10400		52	AHRS
L. Bais, et al. (19)	2014	16	RCT	rTMS	LTPJ	1	90	6	2	14400		240	AHRS, PANSS
		15		rTMS	LTPJ -> RTPJ	1	90	6	2	14400		240	AHRS, PANSS
E. Kim, et al. (17)	2014	22	RCT -Crossover	rTMS	LTPJ	20	100	3	2	12000		60	AHRS
		22		rTMS	LTPJ	1	100	5	2	12000		200	AHRS
		22		rTMS	Broca’s	20	100	3	2	12000		60	AHRS
S. Dollfus, et al. (20)	2018	26	RCT	rTMS	fMRI LSTS	20	80	2	2	10400		52	AHRS, PANSS
J. Brunelin, et al. (14)	2022	22	Cohort	rTMS	LTPJ	1	110	15	2	10800		255	AHRS
X. Liu, et al. (21)	2023	11	RCT	cTBS	LTPJ	50	80	10	8	144000		180.8	PANSS, PSYRATS
P. Tyagi, et al. (22)	2022	30	RCT	cTBS	R TPJ -> LTPJ	50	80	10	2	12000		13.32	PANSS, PSYRATS
L. Marquardt, et al. (30)	2022	11	RCT	tDCS	A - DLPFC, C - L TPJ			5	2		2	200	PANSS
A. Bose, et al. (29)	2018	12	RCT	tDCS	A - DLPFC, C - L TPJ			5	2		2	200	AHRS
		13		tDCS	A - DLPFC, C - L TPJ			5	2		2	200	AHRS
C. Chang, et al. (28)	2018	30	RCT	tDCS	A - DLPFC, C - L TPJ			5	2		2	200	AHRS, PANSS
Y. Yoon, et al. (27)	2019	7	Cohort	tDCS	A - DLPFC, C - L TPJ			5	2		2	200	AHRS, PANSS, PSYRATS
J. Kantrowitz, et al. (26)	2019	47	RCT	tDCS	A - DLPFC, C - L TPJ			5	2		2	200	AHRS, PANSS
Y. Yamada, et al. (31)	2023	28	Cohort	tDCS	A - DLPFC, C - L TPJ			5	2		2	200	PANSS
		15		tDCS	A - STS, C -FP2			5	2		2	200	PANSS
V. Sreeraj, et al. (32)	2018	19	Cohort	tDCS HD	L TPJ			5	2		2	200	PSYRATS
J.P. Lindenmayer, et al. (25)	2018	15	RCT	tDCS	A - DLPFC, C - L TPJ			20	2		2	800	AHRS, PANSS
S. Koops, et al. (24)	2018	28	RCT	tDCS	A - DLPFC, C - L TPJ			5	2		2	200	AHRS, PANSS
J. Mellin, et al. (23)	2019	7	RCT	tDCS	A - DLPFC, C - L TPJ			5	2		2	200	AHRS, PANSS
		8		tACS	A - DLPFC, C - L TPJ	10		5	2		2	200	AHRS, PANSS

A, Anode; C, Cathode; RCT, Randomized Control Trial; LTPJ, Left Temporoparietal Junction; LSTS, Left superior temporal sulcus; DLPFC, Dorsolateral prefrontal cortex; AHRS, Auditory Hallucination Rating Scale; PANSS, Positive And Negative Syndrome Scale; PSYRATS, Auditory Hallucinations Subset of the Psychotic Rating Scale; MT, Motor Threshold.

Of the TMS treatment groups, nine used rTMS (14, 16–20) and two used TBS (21, 22). For guidance of coil localization, nine of the groups used the 10–20 International System of EEG electrode placement (TTIS) and two groups used fMRI guidance (18, 20). This was done by determining the area of maximal activation along the L superior temporal sulcus (STS) while the patient performed a language task. Six of the groups localized to the Left Temporoparietal Junction (L-TPJ), one group to Broca’s area (17, 19), and two groups used a bilateral approach targeting both the Right Temporoparietal Junction (R-TPJ) and L-TPJ (19).

The time periods stretched from 2 days to 15 days. Five treatment groups used high frequency stimulation (20Hz) and four used low frequency (1Hz). The two TBS treatment groups utilized cTBS where pulses are provided in triplets at 50 Hz. All of the rTMS and TBS groups provided a similar total number of bursts, between 10,400 and 14,400, except for X.Liu’s study which administered 144,000 bursts (21). This was made possible by administering eight sessions daily for 10 days, while all other treatment groups used two sessions daily.

Within the ten tES studies evaluated (23–32), there were thirteen treatment groups. Of these groups, eleven used tDCS, one High-Definition tDCS (HD-tDCS) (32), and one used tACS (17, 19, 23). Of the tDCS groups, all but one used the electrode montage of the anode on the Dorsolateral Prefrontal Cortex (DLPFC) and the cathode on the L-TPJ. The tACS group also used this montage. One tDCS group put the anode on the Superior Temporal Sulcus (STS) and the cathode on the L-TPJ. The HD-tDCS group located its electrodes around the L-TPJ (32). All studies used the TTIS. All studies used a BID schedule, and all but one treatment group stretched over 5 consecutive days, accumulating 10 total sessions. The one outlier used a BID schedule for 20 days, totaling 40 sessions. All studies used the intensity of 2mA and had session lengths of 20 minutes.

### Outcome measures

#### Auditory hallucinations

**Figure 4:**
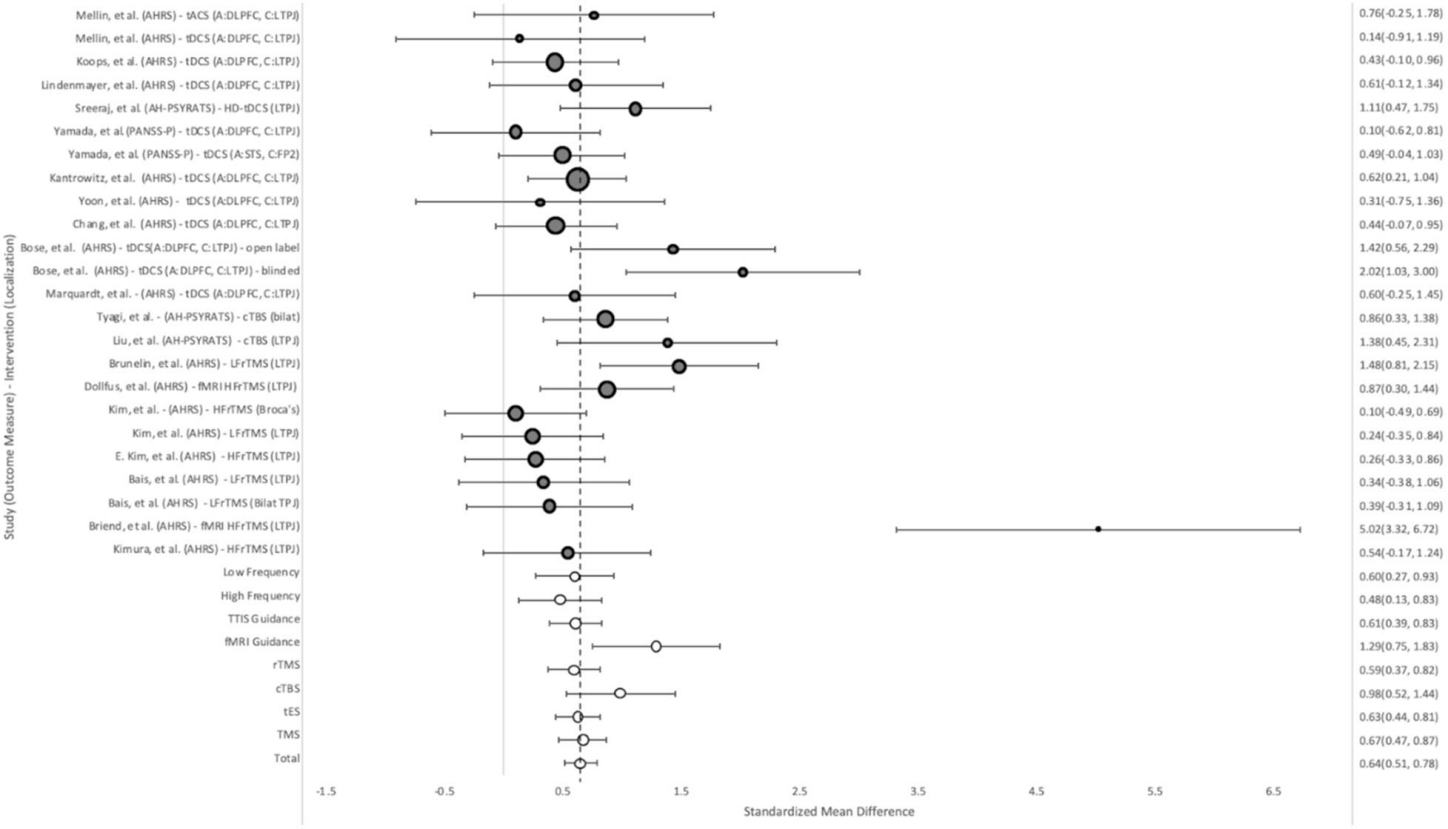
Weighted pooled effect size between AHs before and after treatment with TMS or tES. Dashed line - overall mean improvement. Grey bubbles - effect size of studies. White bubbles - average effect sizes.

AHs were measured using AHRS in 18 treatment groups, AH-PSYRATS in three, and PANSS-P in two. Improvement over time was seen in all studies, regardless of stimulus location or protocol (**Figure 4**). The overall SMD for group-by-time was 0.64 (95%CI = 0.51, 0.78). TMS showed a slightly superior effect to tES, with SMDs of 0.67 (95%CI = 0.47, 0.87) and 0.63 (95%CI = 0.44, 0.81) respectively. Within the subgroups of TMS, the largest improvement in AH was seen in the fMRI guidance groups, which had an effect size of 1.29 (95%CI = 1.75, 0.83), compared to the TTIS guided groups with an SMD of 0.61 (95%CI = 0.83, 0.39). The cTBS groups also showed better than average improvement in AH, with a SMD of 0.98 (95%CI = 1.44, 0.52). Of the groups using the TTIS, those targeting the LTPJ showed more improvement than those targeting Broca’s area or both the L and R-TPJ. High frequency TMS (20 Hz) (0.48, 95%CI = 0.13, 0.83) bursts vs low frequency (1 Hz) (0.60, 95%CI = 0.27, 0.93) bursts, showed similar effects.

**Figure 5:**
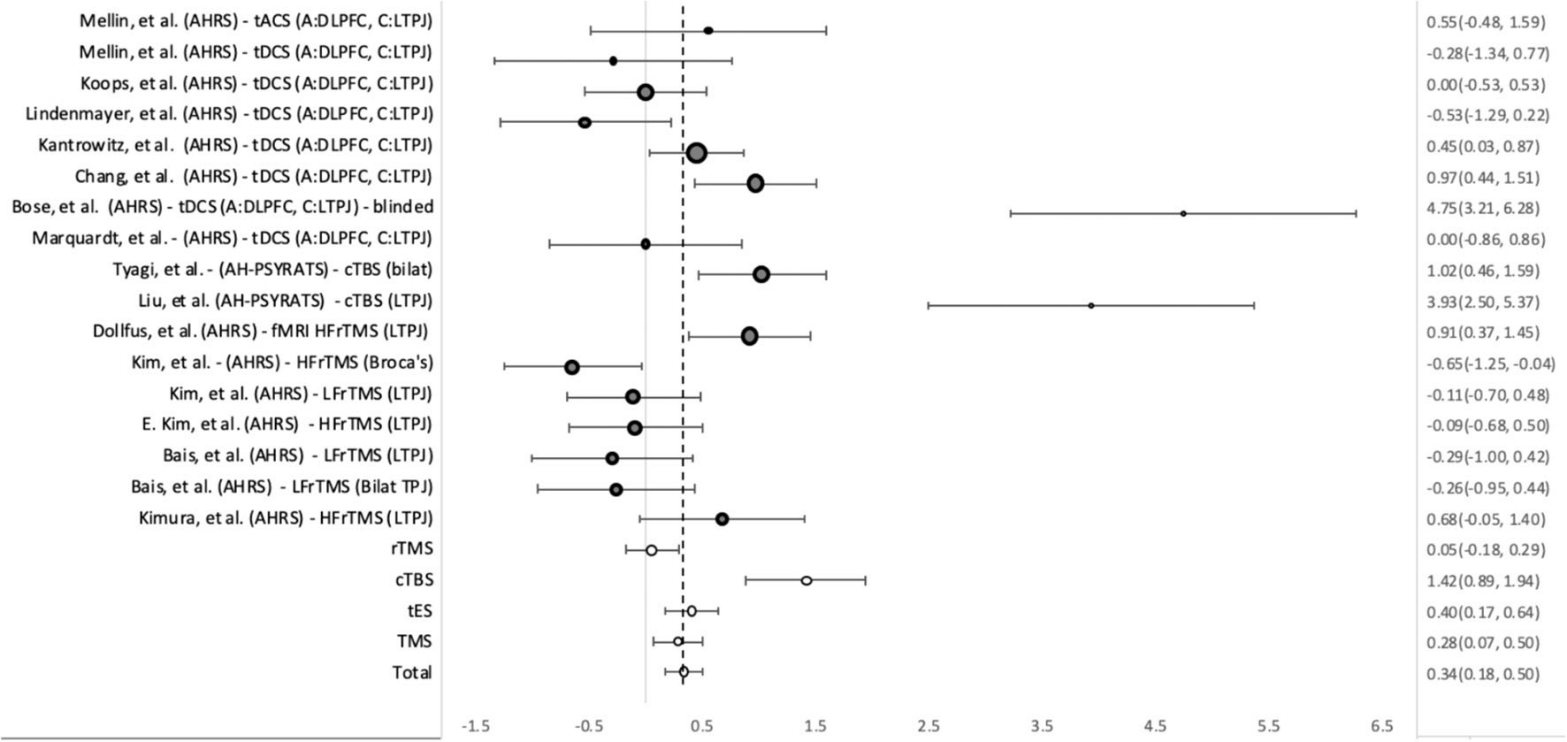
Weighted pooled effect size of improvement of AHS between sham and treatment groups after TMS or tES treatment. Dashed line - overall mean improvement. Grey bubbles - effect size of studies. White bubbles - average effect sizes.

These results were generally consistent with the between-group analysis, with fMRI guidance (0.91, 95%CI = 0.37, 1.45) and cTBS (1.42, 95%CI = 0.89, 1.94) demonstrating the strongest results (**Figure 5**). Overall, tES (0.40, 95%CI = 0.17, 0.64) and cTBS showed statistically significant improvement vs. their sham groups, while rTMS (0.05, 95%CI = -0.18, 0.29) did not. The test for heterogeneity showed significant heterogeneity between the studies (Q = 0.62).

#### Positive symptoms

**Figure 6:**
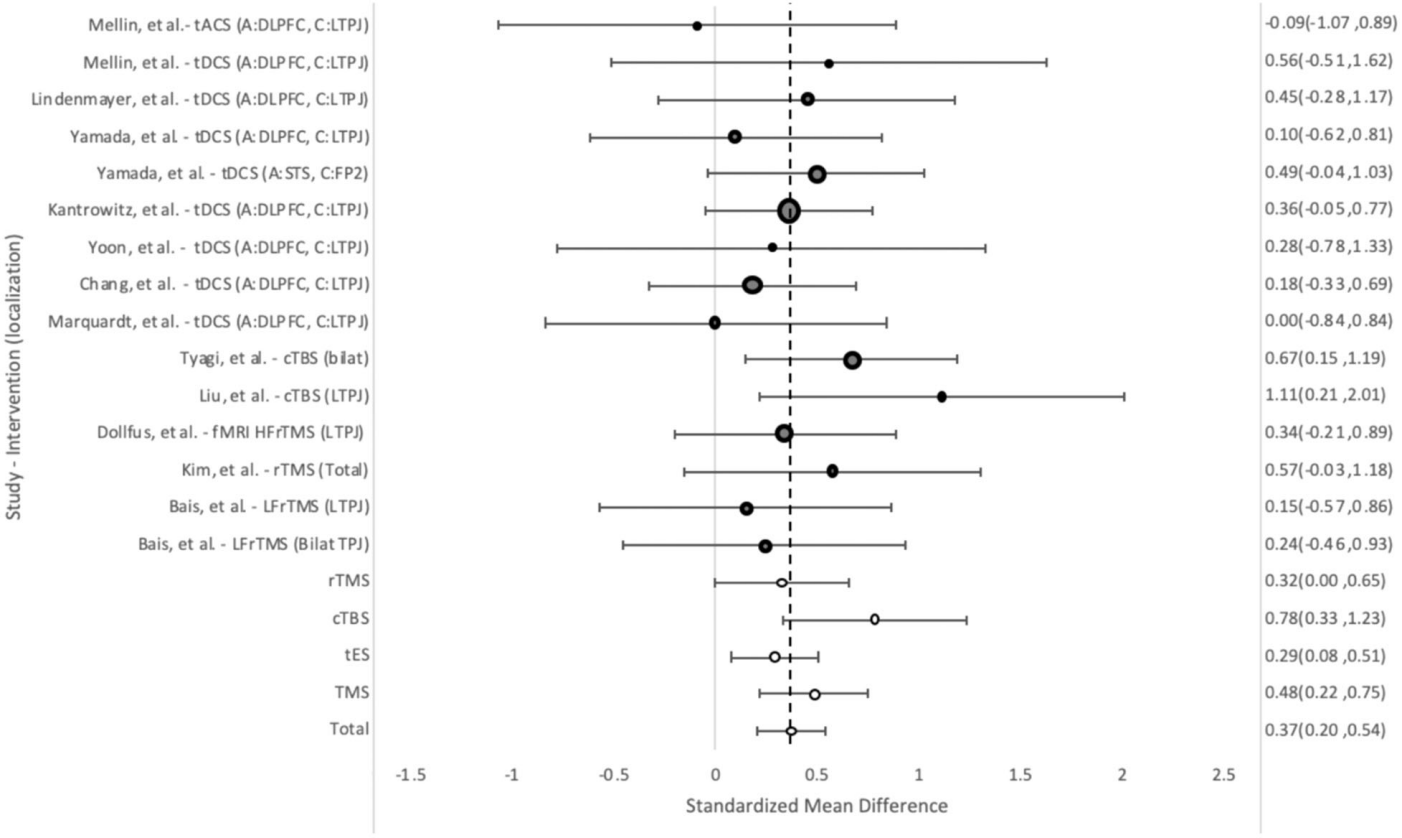
Weight pooled effect size between subjects before and after treatment with rTMS or tES for positive psychotic symptoms as measured by PANSS-P. Dashed line - overall mean improvement. Grey bubbles - effect size of studies. White bubbles - average effect sizes.

The PANSS-P was used to measure change in positive symptoms in 12 treatment groups. Overall, the tES groups (0.29, 95%CI = 0.08, 0.51) performed somewhat worse compared to the rTMS groups (0.48, 95%CI = 0.22, 0.75) (**Figure 6**) in group-by-time analysis. The cTBS groups performed best (0.78, 95%CI = 0.33, 1.23), with X. Liu’s study providing the largest improvement (1.11 95%CI = 0.21, 2.01).

This pattern held for the between-group analysis, with cTBS demonstrating the most improvement (0.78 95%CI = 1.40, 0.90, 1.89) (**Supplemenatry Figure 1**) and neurostimulation overall providing a significant effect (0.62, 95%CI = 0.42, 0.82).

#### Negative symptoms

**Figure 7:**
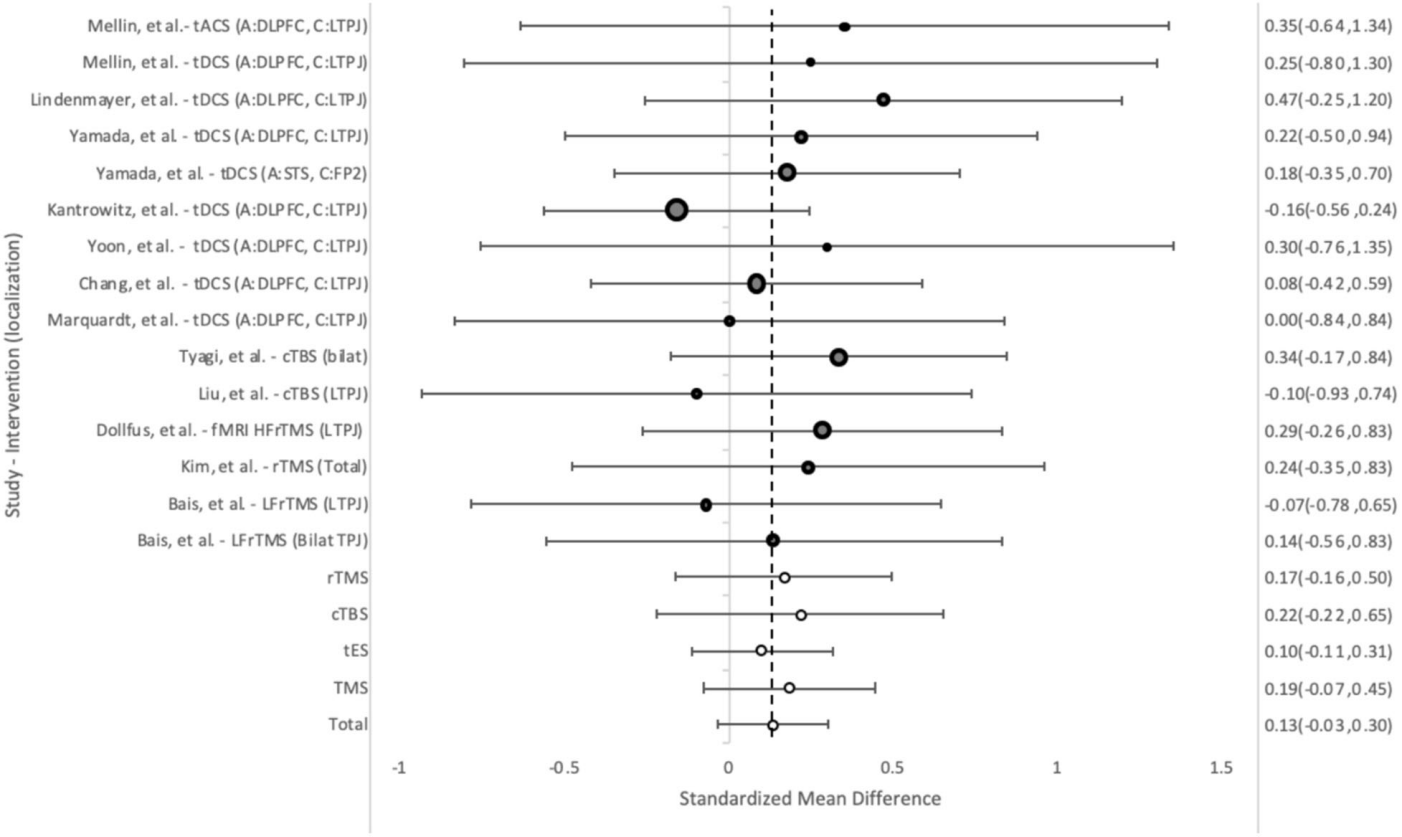
Weighted pooled effect size between subjects before and after treatment with rTMS or tES for negative psychotic symptoms as measured by PANSS-N. Dashed line - overall mean improvement. Grey bubbles - effect size of studies. White bubbles - average effect sizes.

The PANSS-N was used to measure change in negative symptoms in fifteen treatment groups. Of these, eleven groups showed trends toward improvement of negative psychotic symptoms (NPS) in group-by-time analysis, however, none showed statistically significant improvement (**Figure 7**). Overall the effect size was 0.13 (95%CI = 0.30, -0.0 (33)3). TMS trended towards somewhat better results than tES, 0.19 (95%CI= 0.35, -0.08) vs 0.10 (95%CI= 0.31, -0.11).

Similarly, in between-group analysis, no significant improvement was seen when compared to the sham groups overall (0.19,95%CI = 0.00, 0.39), TMS (0.20 95%CI = -0.08, 0.48) or tES (-0.07 95%CI = -0.08, 0.48) (**Supplementary Figure 2**).

## Discussion

This review compares accelerated neurostimulation protocols for the treatment of psychosis. Unlike previous meta-analyses (10, 12, 34, 35), the present study focused specifically on accelerated protocols, defined as protocols that provided neurostimulation more than once per day. Our analysis showed that both TMS and tES protocols improved AHs and PPS, while not meaningfully changing NPS. TMS protocols had a more pronounced but similar effect to tES overall. This aligns with prior research of non-accelerated protocols showing consistent improvement for patients treated with rTMS (35), whereas tDCS has shown more modest or mixed results (34).

For AHs, the rTMS groups that used fMRI guidance showed by far the most improvement. This is in line with prior research, suggesting the superiority of localization using fMRI vs TTIS (33). Cap-based targeting, as is done in TTIS, has been shown to decrease the accuracy and precision of targeting DLPFC in TMS (36). Due to the impracticality of fMRI guidance, the clinical setting typically relies on cap-based methods.

Localization of sites other than the L-TPJ, like the bilateral TPJs, STS, and Broca’s area showed similar or worse responses. This is in line with current research that prioritizes the L-TPJ (37) as it has been consistently demonstrated to play a role in the physiopathology of AHs (38, 39).

High and low frequency rTMS performed roughly the same, with a small priority for low frequency. Prior research has been mixed with which approach is more efficacious, with superiority typically being seen in low frequency rTMS (3, 35). Studies using high frequency bursts were able to shorten the total treatment duration, often to only two or three days in total, without decreasing the total number of bursts provided. This provides another possible opportunity, along with the accelerated scheduling, to expedite treatment outcomes.

cTBS demonstrated a stronger effect on AHs than TMS overall. cTBS, like low Frequency rTMS, produces a Long-Term Depression (LTD) effect on neurons rather than a Long Term Potentiation (LTP) effect like iTBS and HF-rTMS, possibly pointing to why these protocols have yielded the most benefit (40). TBS protocols are shorter and more efficient than TMS as they provide stimulation at a much higher frequency. X. Liu’s Study provided roughly ten times the total number of pulses as the other TMS studies, while providing treatment for 59 fewer minutes than the LF-rTMS studies. This is especially important considering a dose dependent relationship has been demonstrated for these interventions (10, 41).

TBS has also shown promise as a tool for the use of other positive and negative symptoms of schizophrenia, especially considering its strong effects on neuronal plasticity (42). In this study cTBS groups elicited the strongest response to PPS, but no clear effect on NPS.

Regarding NPS, all of the treatment types yielded only modest improvements, with very little difference between one another. This may be because the stimulus locations were focused on improving AH. Other intervention sites like the DLPFC have been utilized in the past in order to improve negative symptoms more substantially (43). This also may be because many of the treatment groups, ie LF-rTMS or cTBS, provided an inhibitory stimulus. Excitatory neurostimulation, like in the case of iTBS, has shown potential (10).

tDCS studies were more consistent in their approach, generally placing the anode over the DLPFC and the cathode over the left TPJ, with a twice-daily (BID) stimulation schedule over multiple consecutive days. One study utilizing transcranial alternating current stimulation (tACS) showed results comparable to the tDCS group as a whole.

Overall, this study points to accelerated approaches having a comparable effect on AH and PPS to non-accelerated approaches. The SMD of 0.34 yielded in the between-group analysis of AHs suggests a small to moderate effect size. Previous meta-analyses of non-accelerated protocols have yielded comparable effect sizes (10, 44) including 0.29 (95%CI = 0.02, 0.57) (6) for LFrTMS, 0.44(p <0.01) (45) for rTMS, and 0.50 (95%CI -0.09,1.09) for tDCS (12).

There are many limitations to this study. First, the heterogeneity in stimulation protocols and outcome measures among the studies complicates direct comparisons. Furthermore, many of the studies evaluated are single-arm studies or have small sample sizes which limits the generalizability of these findings. Additionally, the metaanalysis contains data from both cohort and RCT studies, which may yield different results. Another limitation is that this study only reviewed one database and did not include data from case studies or from studies published before 2014, meaning it is not an exhaustive look at the data.

Future research should prioritize randomized controlled trials with standardized protocols and larger sample sizes to better understand the optimal way to deliver these interventions. Direct comparisons in efficacy and practicality between accelerated and non-accelerated protocols would also be very useful. Additionally, long-term studies are needed to assess the sustainability of these effects and potential adverse outcomes, ultimately informing clinical practices and improving patient care.

## Data Availability

The original contributions presented in the study are included in the article/**Supplementary Material**. Further inquiries can be directed to the corresponding author.
