# Supplementary figures and images for: Accelerated neurostimulation protocols for auditory hallucinations: a systematic review and meta-analysis
Source: Front Psychiatry. 2025 Jun 13;16:1491487. doi: 10.3389/fpsyt.2025.1491487 (PMC12202435; doi:10.3389/fpsyt.2025.1491487)

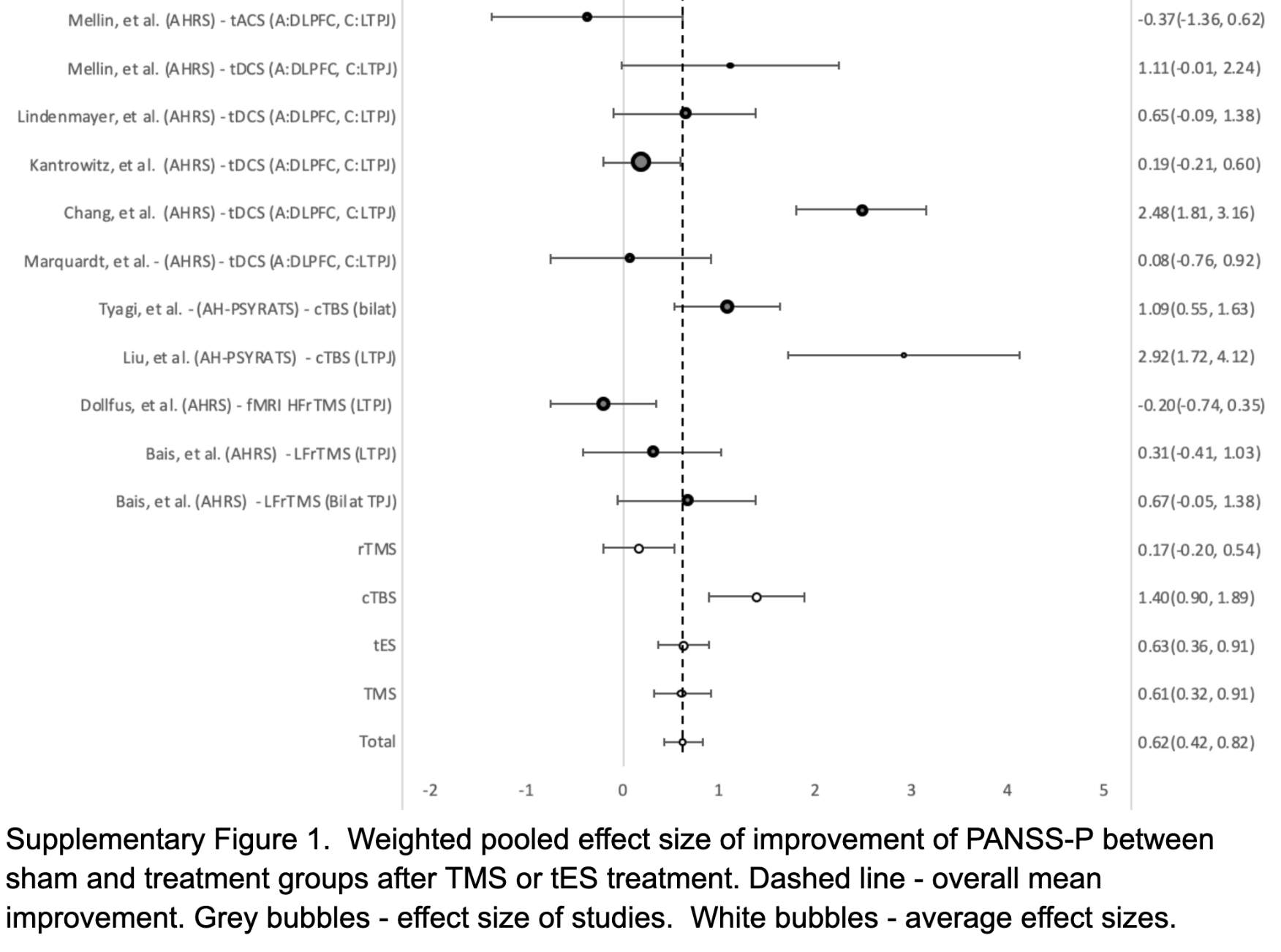

Supplement: Supplementary file 1 [file Image1.tiff]

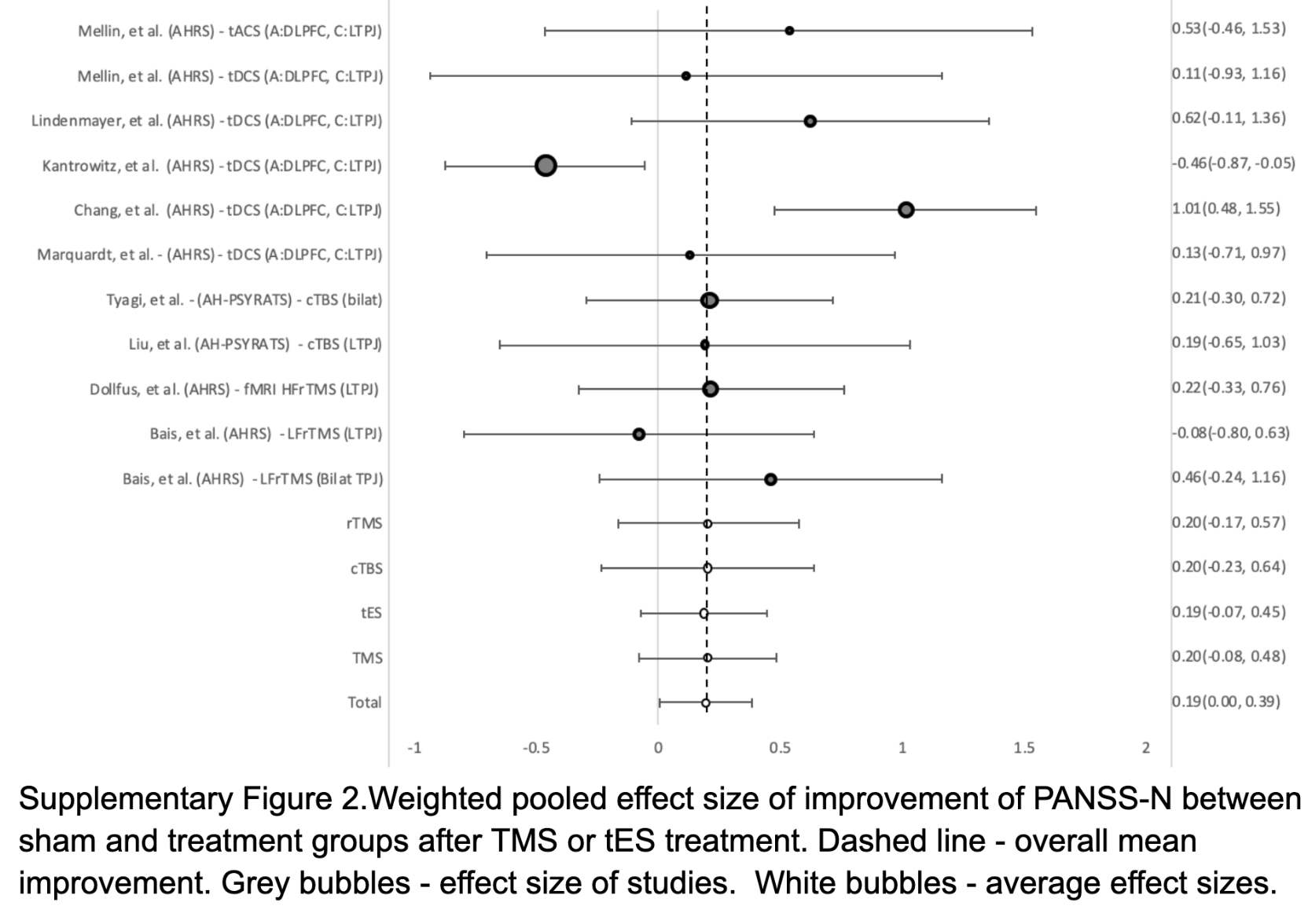

Supplement: Supplementary file 2 [file Image2.tiff]
